# A Novel Method for Localizing PD Source in Power Transformer: Considering NLOS Propagation of Electromagnetic Waves

**DOI:** 10.3390/s25165099

**Published:** 2025-08-16

**Authors:** Qingdong Zhu, Mengzhao Zhu, Wenbing Zhu, Chao Gu, Cheng Pan, Zijun Pan

**Affiliations:** 1 State Grid Shandong Electric Power Research Institute, Jinan 250003, China; 2 School of Electrical Engineering and Automation, Wuhan University, Wuhan 430072, China

**Keywords:** partial discharge, transformers, ultra-high-frequency localization, time difference in arrival, non-line-of-sight

## Abstract

A novel partial discharge (PD) source localization method was proposed based on the traditional time difference in arrival (TDOA) method. Specifically, the non-line-of-sight (NLOS) propagation phenomenon of the ultra-high-frequency (UHF) signal was considered, and the NLOS propagation error was approximately replaced by a constant, thereby limiting the effect of NLOS propagation. Moreover, the strategy of utilizing more than four sensors was adopted to reduce the possible effect of overcorrection on NLOS propagation. In this paper, the derivation and implementation process of the proposed method is introduced from the perspectives of mathematical model and geometrical model, and its localization results were compared with the traditional TDOA method through an experimental study. The results showed that the speed of error increase of the traditional method presented faster, and the increment of sensor number helped to improve the localization accuracy, but the reduction in localization error becomes insignificant when the sensors exceed six. Finally, the experimental verifications were conducted based on a 35 kV testing transformer with six sensor installations. The experiments found that the proposed localization method had a better calculated accuracy and stability; the obtained minimum calculated error was 10.88 cm, the calculated accuracy can be improved by 82.04% and 78.94%, respectively, with six sensors than four and five sensors arrangement.

## 1. Introduction

As one of the most crucial equipment in the power system, the insulation reliability of large power transformers is related to the safe and stable operation of the transformer itself and even the whole power system [1,2]. During long-term operation, power transformers often experience insulation damage due to partial discharge (PD), which can ultimately lead to insulation degradation or even failure. However, PD characteristics can serve as indicators of insulation condition and are commonly employed for insulation detection and condition diagnosis purposes [3,4]. The ultra-high frequency (UHF) method is widely used in PD detection due to its advantages of high sensitivity, strong anti-interference ability, and non-contact measurement [5,6]. Moreover, the target signal detected by the UHF method is the electromagnetic wave generated by partial discharge, and the propagation characteristics of electromagnetic waves will change due to their location in power equipment; thus, the UHF method can also be used for localizing the position of the partial discharge source [7].

The time difference in arrival (TDOA) method has emerged as a prominent method for locating UHF PD sources due to its high accuracy and simple principle [8,9]. This method assumes that the electromagnetic wave propagates along a straight line, and four UHF sensors are used to collect the PD signals at the same time. Afterwards, the signal acquired by one of the sensors is taken as the reference, and the time delay between the remaining three signals and the reference signal can be calculated. Finally, the 3D spatial coordinates of the PD source position can be obtained by solving the hyperbolic localization equations [10,11].

It can be seen that the conventional TDOA method presents relatively high accuracy in locating PD sources in GIS. However, when it is applied to large power transformers, due to the existence of the core, windings, and other structures will block the electromagnetic wave signals so that they cannot be propagated along a straight line [12]; the TDOA localization results will be a great deviation, or even positioning failure.

The TDOA method has been widely recognized for its accuracy in localizing PD sources in equipment like GIS with simple internal structures [13]. However, it still faces difficulties when applied to large power transformers due to the existence of complex inter-elements such as the core and windings. These elements obstruct the straight-line propagation of electromagnetic waves, leading to the so-called non-line-of-sight (NLOS) propagation phenomenon of the signal, which may cause significant deviations in the localization results or even localization failures. Du et al. investigated the impact of the iron core and winding on the propagation time of electromagnetic wave signals in a 110 kV real transformer. The results demonstrated that the measured propagation time was much larger than the straight-line propagation time when the PD source and the UHF detection antenna were separated by the presence of an iron core and winding, and the error can be up to more than 5 ns [14]. In response to the problem of low positioning accuracy caused by the NLOS propagation of the ultrasonic signals, Jia et al. proposed a PD source localization method for transformers under the condition of complex multipath signal propagation, which provided a solution to the localization problem of electromagnetic wave signals in NLOS propagation scenarios [15]. However, due to the propagation of electromagnetic wave signals being more than five orders of magnitude faster than that of ultrasonic signals, resulting in a smaller time delay, the impact of the NLOS propagation phenomenon on localization accuracy should become more severe.

Currently, no effective strategy exists to address the issue of diminished location accuracy caused by NLOS propagation of electromagnetic waves. Therefore, this paper proposes a method for localizing the PD source position in transformers while considering NLOS propagation effects. Firstly, a mathematical model was constructed to correct the positioning deviation caused by NLOS propagation, and an analysis of the geometric framework underlying the model was conducted. In addition, the strategy of utilizing more than four UHF sensors was proposed to enhance localization accuracy. Finally, the feasibility and efficacy of the proposed method were verified through both lab and on-site experiments.

## 2. Localization Model of Transformer Partial Discharge Source

### 2.1. Mathematical Model

**Figure 1:**
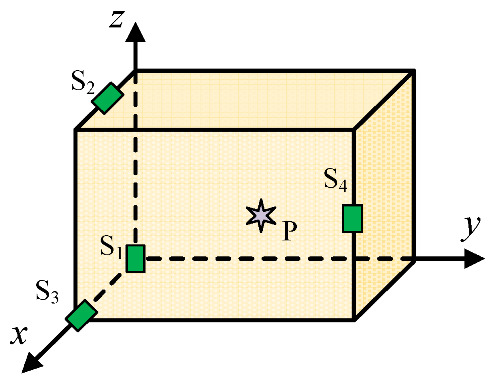
The coordinate system of the PD source localization model.

A three-dimensional coordinate system is established with one corner of the transformer serving as the spatial origin, as shown in Figure 1. Here, the power transformer is simplified as a rectangular box. *P* represents the PD source, while *S* denotes UHF sensors installed around the transformer.

The three-dimensional spatial coordinates of *P* and each UHF sensor S*_i_* are expressed as *P* (*x_P_*, *y_P_*, *z_P_*) and *S_i_* (*x_i_*, *y_i_*, *z_i_*) (*i* = 1, 2, …, *N*), respectively. The distance along which the electromagnetic wave propagates from *P* to *S_i_* can be represented by the following equation:

(1) $$r_{i}=ct_{i}=d_{i}+b_{i}+n_{i}$$

Here, *r_i_* represents the actual distance for the electromagnetic wave signal propagating from *P* to *S_i_*. *c* denotes the speed of the electromagnetic wave propagation, and *t_i_* indicates the time for the electromagnetic signal to travel from *P* to *S_i_*. Sensor 1 is established as the reference sensor. For N sensors, Sensor 1 is established as the reference sensor, and *t_1_* is the propagation time for the electromagnetic wave signal from *P* to *S*_1_. Therefore, the propagation time *t_i_* for the sensor *S_i_* can be expressed in terms of *t*_1_ and the signal time delay Δ*t*_1*i*_:

(2) $$t_{i}=t_{1}+\Delta t_{1i}$$

Based on (1), *r_i_* can be represented by three components: *d_i_*, *b_i_*, and *n_i_*. Where *d_i_* denotes the straight line distance from *P* to *S_i_*, and can be expressed as follows:

(3) $$d_{i}=\sqrt{{\left(x_{i}-x_{P}\right)}^{2}+{\left(y_{i}-y_{P}\right)}^{2}+{\left(z_{i}-z_{P}\right)}^{2}}$$

In addition, *b_i_* represents the propagation distance error induced by NLOS phenomena such as reflection, refraction, and diffraction of electromagnetic wave signals. When the electromagnetic wave signals undergo NLOS propagation inside the transformer, the propagation path is correspondingly elongated. Consequently, this error component manifests as a positive deviation, that is, *b_i_* ≥ 0.

Here, *b_i_* denotes the measurement error due to Gaussian random noise, and it is generally considered that *n*_i_ obeys a Gaussian distribution with a mean of zero and a variance of *σ*^2^ [16]. Therefore, the problem of localizing the coordinates of the PD source can be reformulated as the following Maximum Likelihood Estimation (MLE) problem:

(4) $$\underset{x_{P},y_{P},z_{P}}{\arg\;\min}\sum_{i=1}^{N}\frac{{\left(r_{i}-d_{i}-b_{i}\right)}^{2}}{\sigma^{2}}$$

It can be seen that, if *b_i_* is known, the (4) possesses a unique solution. However, the specific propagation path of the electromagnetic wave signal inside the transformer is unknown, and the distribution of *b_i_* cannot be obtained. If *b_i_* = *r_i_* − *d_i_*, (4) can have an infinite number of solutions. In order to make the problem solvable, a constant $\hat{b}$ is used here to approximate bi instead, thereby (4) is transformed into

(5) $$\underset{x_{P},y_{P},z_{P}}{\arg\;\min}\sum_{i=1}^{N}\frac{{\left(r_{i}-d_{i}-\hat{b}\right)}^{2}}{\sigma^{2}}$$

The method of employing a constant $\hat{b}$ as an approximation for *b_i_* will inevitably result in a decrease in localization accuracy, where the value of $\hat{b}$ exerts a direct influence on this accuracy. Selecting a suitable value of $\hat{b}$ is the critical point to actualize the PD source localization. To quantitatively evaluate the substitution effect of $\hat{b}$ value, the approximation error *ρ* is defined as follows:

(6) $$\rho ={\left[\sum_{i=1}^{N}\frac{{\left(r_{i}-d_{i}-b_{i}\right)}^{2}}{\sigma^{2}}-\sum_{i=1}^{N}\frac{{\left(r_{i}-d_{i}-\hat{b}\right)}^{2}}{\sigma^{2}}\right]}^{2}$$

In general, the NLOS propagation error has a much more serious effect on the localization results compared to the Gaussian random error, i.e., *b_i_* >> *n_i_*, then (6) can be expressed as

(7) $$\rho \approx {\left[\sum_{i=1}^{N}\frac{{\left(b_{i}-\hat{b}\right)}^{2}}{\sigma^{2}}\right]}^{2}$$

A smaller *ρ* implies that the chosen value of $\hat{b}$ provides a more effective approximation, thereby minimizing the decline in localization accuracy. The optimal value of $\hat{b}$ is attained when (7) equals zero, that is,

(8) $$\begin{array}{ll} & \hat{b}=\arg\min{\left[\sum_{i=1}^{N}\frac{{\left(b_{i}-\hat{b}\right)}^{2}}{\sigma^{2}}\right]}^{2}\\ & \;=\arg\min\sum_{i=1}^{N}\frac{{\left(b_{i}-\hat{b}\right)}^{2}}{\sigma^{2}}\\ & \;=\sum_{i=1}^{N}\frac{b_{i}}{N} \end{array}$$

However, since the distribution of *b_i_* is unknown, it is still hard to ascertain the optimal value of $\hat{b}$. Nevertheless, given that *r_i_* = *d_i_* + *b_i_* + *n_i_* > *b_i_*, the upper bound of $\hat{b}$ can be presented as

(9) $$\hat{b}<\sum_{i=1}^{N}\frac{r_{i}}{N}$$

Note that a certain geometrical relationship should also be satisfied between the sensors *S_i_*, *S_j_*_,_ and the PD source:

(10) $$c(t_{1}+\Delta t_{1i})+c(t_{1}+\Delta t_{1j})=r_{i}+r_{j}\geq d_{i}+d_{j}\geq d_{ij}$$

Substituting (2), (3), (9), and (10) as constraints into (5), the problem of localizing the PD source can be rewritten as

(11) $$\begin{array}{l} \underset{t_{1},x_{P},y_{P},z_{P},\hat{b}}{\arg\;\min}\sum_{i=1}^{N}\frac{{\left(r_{i}-d_{i}-\hat{b}\right)}^{2}}{\sigma^{2}}\\ s.t.\;r_{i}=c(t_{1}+\Delta t_{i1})\\ \;d_{i}=\sqrt{{\left(x_{i}-x_{P}\right)}^{2}+{\left(y_{i}-y_{P}\right)}^{2}+{\left(z_{i}-z_{P}\right)}^{2}}\\ \;\hat{b}<\frac{\sum_{i=1}^{N}r_{i}}{N}\\ \;c(t_{1}+\Delta t_{1i})+c(t_{1}+\Delta t_{1j})=r_{i}+r_{j}\geq d_{i}+d_{j}\geq d_{ij} \end{array}$$

As seen, (11) is an objective function to describe the nonlinear optimization problem, and in order to transform it into an unconstrained optimization problem, an external penalty function [17] is added to the objective function (11) here. The constraints involving *t*_1_ and $\hat{b}$ are integrated into the original objective function, substantially increasing the value of the objective function for points that do not satisfy Equations (9) and (10), while leaving the objective function value unchanged for points that do satisfy these equations. Thus, (11) is reformulated as

(12) $$\begin{array}{l} \underset{(x_{s},y_{s},z_{s}),\hat{b},t_{1}}{\min}\sum_{i=1}^{N}\frac{{\left(r_{i}-d_{i}-\hat{b}\right)}^{2}}{\sigma^{2}}+M\times [\max(0,\hat{\beta }-\frac{\sum_{i=1}^{N}r_{i}}{N})\\ +\sum_{i\neq j}\max(0,\frac{d_{ij}-c(\Delta t_{1i}+\Delta_{1j})}{2c}-t_{1})]\\ s.t.d_{i}=\sqrt{{\left(x_{s}-x_{i}\right)}^{2}+{\left(y_{s}-x_{i}\right)}^{2}+{\left(z_{s}-z_{i}\right)}^{2}} \end{array}$$

### 2.2. Geometrical Model

Based on the mathematical form of localization equations, it needs at least four sensors to localize the PD source *P* within the three-dimensional space.

**Figure 2:**
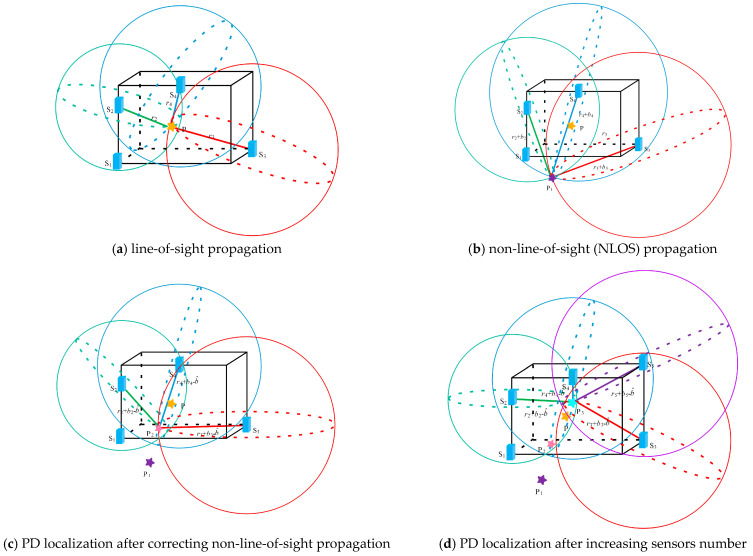
Geometric schematic of PD source localization.

Taking four sensors as an example, if S_1_ is the reference sensor, its time delays between sensors S_2_, S_3_, and S_4_ are Δ*t*_12_, Δ*t*_13_, and Δ*t*_14_ respectively. are the time delays. Then the radii *r*_d2_, *r*_d3_, and *r*_d4_ of the localization circle are determined by combining the propagation time *t*_1_ for the electromagnetic wave signal to travel from P to S_1_. When there is no measuring error in Δ*t*_12_, Δ*t*_13_, and Δ*t*_14_, the localization circles will intersect at the actual position of the PD source, as illustrated in Figure 2a.

However, the distance errors *b*_2_, *b*_3_, and *b*_4_ introduced by NLOS propagation will increase the length of *r*_d2_, *r*_d3_, and *r*_d4_. As a consequence, the intersection point of the localization circles will not coincide with the actual position of the PD source, and may even fall outside the transformer, leading to localization failure, as shown in Figure 2b. And it should be noted that it is hard to correct the propagation path of electromagnetic wave signals by gradually reducing the localization circle radius when *b*_2_, *b*_3_, and *b*_4_ are unknown.

Based on Equation (5), if a constant $\hat{b}$ is used to substitute for *b*_2_, *b*_3_, and *b*_4_, the radius of each localization circle would be reduced by an equal amount, which leads to a decrease in localization accuracy. According to (12), the value of $\hat{b}$ is calculated, and its minimum is attained where the sum of the squared distances to each localization circle is the smallest, as illustrated in Figure 2c.

Moreover, it can be seen from (9) that $\hat{b}$ only has an upper limit, so when the number of sensors is small, the radius reduction range of the localization circle will become large, which may result in an overcorrection on NLOS propagation errors. Thus, to further improve the localization accuracy, the strategy of increasing the number of sensors should be adopted. It is because the increased number of sensors can narrow the range of $\hat{b}$, suppressing the overcorrection of errors introduced by NLOS propagation, as shown in Figure 2d.

### 2.3. Localization Process of PD Source

**Figure 3:**
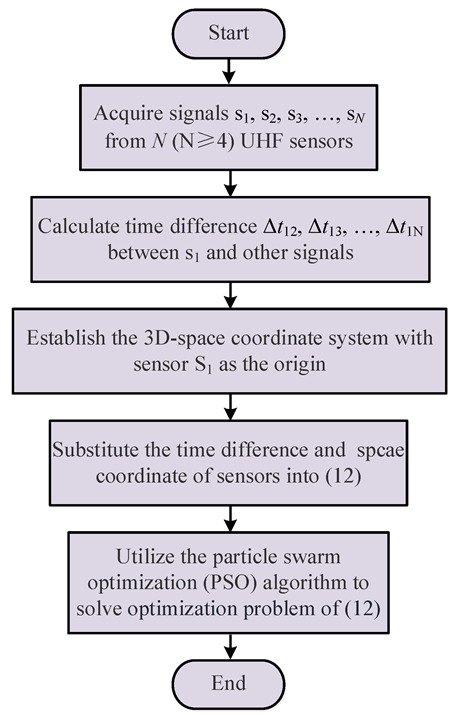
The localization process of PD source.

The localization process of PD source is shown in Figure 3, and the detailed description are as follows:

(1) The N (N ≥ 4) UHF sensors S_1_, S_2_, S_3_, …, S_N_, placed in random positions around the PD source, are utilized to simultaneously collect PD signals s1, s2, s3, …, sN.

(2) Taking s1 as the reference signal, the signal time difference Δt_12_, Δt_13_, …, Δt_1*N*_ between s1 and s2, s3, …, sN are calculated, respectively, by the energy accumulation method [18], and the calculated results are then substituted into (12).

(3) Taking the sensor S1 as the origin, a 3D spatial coordinate system is established. The coordinates (xi, yi, zi) of the sensor S_i_ (where i = 1, 2, …, N) are then substituted into (12).

(4) To solve the optimization problem of (12). Here, the Particle Swarm Optimization (PSO) algorithm is employed, and the obtained solution (xs, ys, zs) is the 3D spatial coordinates of the PD source.

## 3. Experimental Study on Factors Influencing Localization Accuracy

### 3.1. Experimental Platform

**Figure 4:**
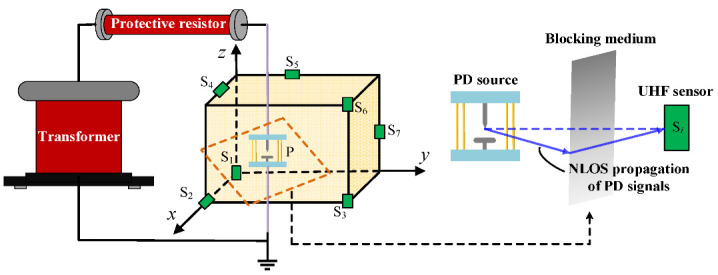
Experimental platform of UHF PD localization.

In order to study the calculated accuracy of the localization method under the influence of NLOS propagation of the signal, an experimental platform of UHF PD localization was established, as shown in Figure 4. The localization test area was set up as a cube space with a side length of 300 cm, and a total of seven sensors were arranged in the space. The coordinates of the sensors were as follows: S_1_ (0, 0, 0), S_2_ (300, 0, 0), S_3_ (300, 300, 0), S_4_ (150, 0, 300), S_5_ (0, 150, 300), S_6_ (300, 300, 300), and S_7_ (0, 300, 150), while the coordinate of PD source was (246, 203, 42). The test circuit consists of a transformer (with a rated voltage of 100 kV and a capacity of 50 kVA), a protective resistor (5 kΩ), and an insulating defect simulated by a pair of needle-plate electrode structures.

Moreover, to simulate NLOS propagation of the electromagnetic wave, an iron sheet (10 cm × 100 cm, thickness 1 cm) was directly attached in front of the selected UHF sensor, as shown in Figure 4. The frequency range of PD UHF signals mainly spans from 300 MHz to 1.5 GHz [19,20], corresponding to a wavelength λ range of approximately 0.2–1 m. In the 300 MHz–1 GHz range, the 10 cm width is generally smaller than λ/2, so the blocking effect is relatively weak, and the electromagnetic wave bypasses the sheet, thus simulating NLOS propagation. The metallic sheet was placed directly against the sensor front, so the direct wave is almost entirely obstructed, and the received signal is dominated by diffracted and reflected components.

### 3.2. The Effect of NLOS Propagation of UHF Signals on Localization Accuracy

**Figure 5:**
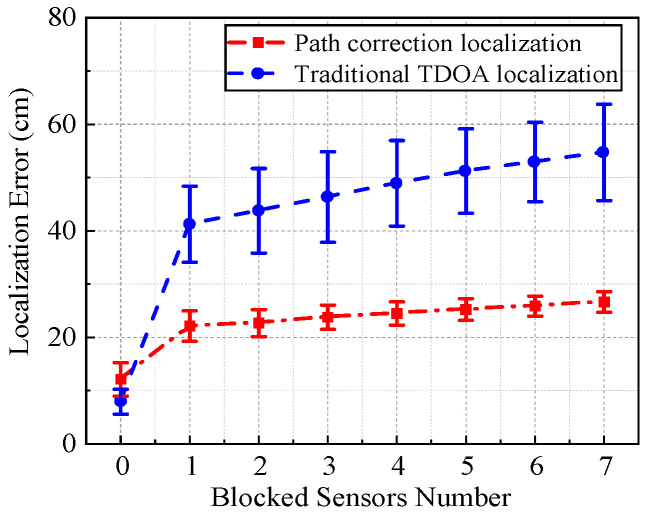
The effect of the number of blocked sensors on localization accuracy.

In this section, all seven sensors were involved in the localization process, and the difference in localization results between the proposed method and traditional TDOA method was compared by altering the number of sensors blocked by the medium, as shown in Figure 5. The Euclidean distance between the localization result and the actual PD source was calculated as the localization error. The smaller the localization error, the better the accuracy of the localization method. It should be noted that when the number of blocked sensors is unchanged, the error bars can be calculated by multiple groups of localization.

It can be seen that when there were no blocked sensors, the localization error of the traditional TDOA localization method was relatively small due to the absence of NLOS propagation. However, the proposed path correction method exhibited a slightly higher localization error because of the overcorrection of NLOS propagation. The localization deviation was only about 20 cm. With the increase in the number of blocked sensors, the localization errors of these two methods tended to gradually increase, and the error of the traditional TDOA localization method grew significantly faster than that of the path correction method. It can be concluded that the NLOS path correction method is superior when there exists one or more blocked sensors.

### 3.3. The Effect of the Number of Sensors on the Localization Accuracy of the NLOS Path Correction Method

**Figure 6:**
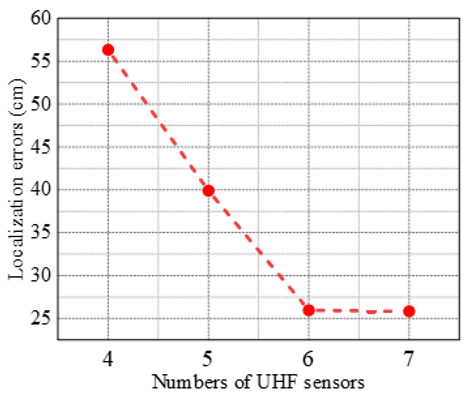
The effect of sensor numbers on localization accuracy of NLOS path correction method.

Moreover, in order to discuss the effect of sensors’ number on the localization accuracy of the NLOS path correction method and to ensure a suitable number of involved UHF sensors, here the localization error was calculated with different numbers of sensors, as illustrated in Figure 6, and all of the involved sensors were blocked by the medium. It can be seen that the localization error decreased significantly during the process of increasing the sensor number from four to six, i.e., from 56.32 cm to 25.83 cm. However, when the number of sensors exceeded six, the rate of reduction in localization error became relatively slow. The probable reason for this is that at this point the upper limit of $\hat{b}$ no longer decreases, but converges to some fixed value, so that there is no longer a significant improvement in positioning accuracy.

From the above analysis, it can be found that, as the number of blocked sensors increases, the localization errors of both the traditional TDOA method and the NLOS path correction method will increase. In addition, the traditional TDOA method can only maintain higher localization accuracy when all sensors are unblocked. When any sensor is obstructed, the localization accuracy will be significantly reduced, and the localization performance becomes inferior to the NLOS method. Due to the complex internal structure of actual power transformers, there are unavoidable obstructions between the internal PD source and the UHF sensors mounted on the enclosure. So the NLOS path correction localization method presents a more accurate localization. Moreover, increasing the number of sensors involved is conducive to further improving the localization accuracy.

## 4. Experimental Verification and Results

**Figure 7:**
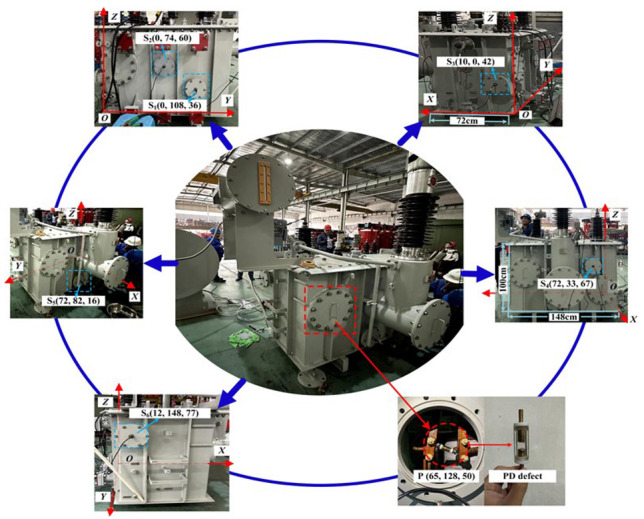
The photo of the testing transformer and the positions of each UHF sensor from a different perspective.

To verify the reliability of the proposed PD localization method, a verification experimental platform was established based on a testing oil-immersed transformer (YOD-500/35) manufactured by Shenda Electric Group Co.,Ltd. from Zhejiang Province, China. The capacity and rated voltage of the transformer are 500 kVA and 35 kV, respectively, and the size of the transformer is 72 cm × 148 cm × 100 cm. As shown in Figure 7, one of the transformer corners was selected as the origin, and then the spatial coordinate can be built. Here, six UHF sensors were installed inside the transformer, and the coordinates of the sensors were S_1_ (0, 108, 36), S_2_ (0, 74, 60), S_3_ (10, 0, 42), S_4_ (72, 33, 67), S_5_ (72, 82, 16), S_6_ (12, 148, 77). In addition, the PD defect was made of an air gap between the rod–rod electrodes, and it was also installed into the transformer, as shown in Figure 7. The position of the PD defect was P (65, 128, 50). Note that all of the units of coordinates are cm.

**Figure 8:**
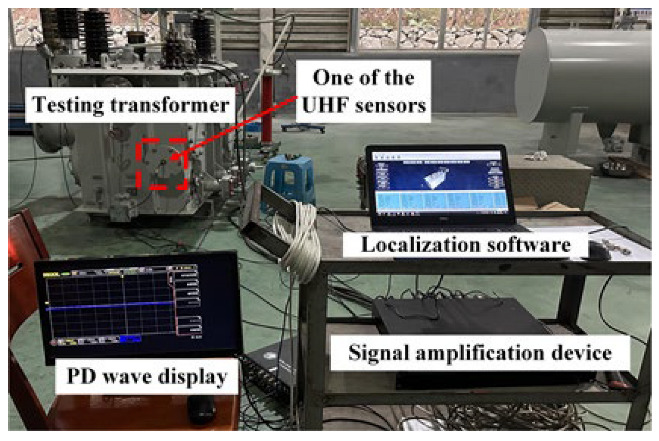
Verification platform of transformer PD localization.

The layout of the verification platform is shown in Figure 8, and its elements contained a testing transformer with six UHF sensors installed, a localization software system, a signal amplification device, and a PD wave display.

**Figure 9:**
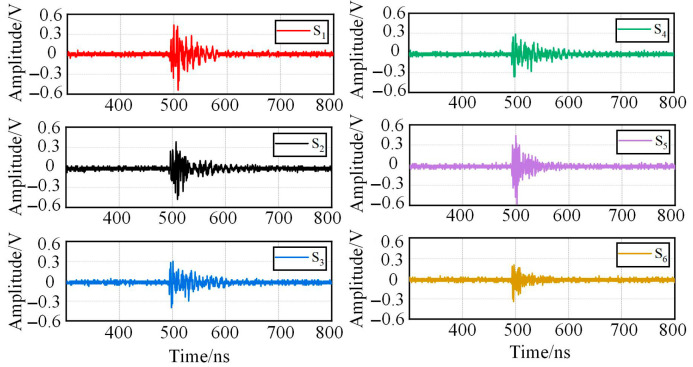
One of the detected results of PD signals from six sensors.

Once the PD occurs, the sensors will capture original UHF signals, then deliver these UHF signals into the signal amplification device, thus the original PD signal can be amplified, and meanwhile, the wave of PD signal can be shown on the screen of PD wave display, one of the detection results of PD wave is shown in Figure 9.

Due to the small size of the transformer in this experiment, the received PD signal amplitude of each sensor was significant. Under this condition, the localization method proposed in this paper can be used to determine the partial discharge location. The amplified signals will be input into the localization software. Here, the proposed NLOS path correction method was packaged into the localization software, and all calculations followed the process in Figure 3.

Here, the Sensor S_1_ was selected as the reference sensor, and the calculated accuracy of the proposed PD localization method was discussed based on the following two parts:

### 4.1. Comparison Between the Traditional TDOA Method

In this part, the PD signals from six sensors were utilized, and then the coordinate of the PD source was calculated by the traditional TDOA method and NLOS path correction method, respectively. The calculated results and localization errors were obtained from three times of experiments, as shown in Table 1.

**Figure 10:**
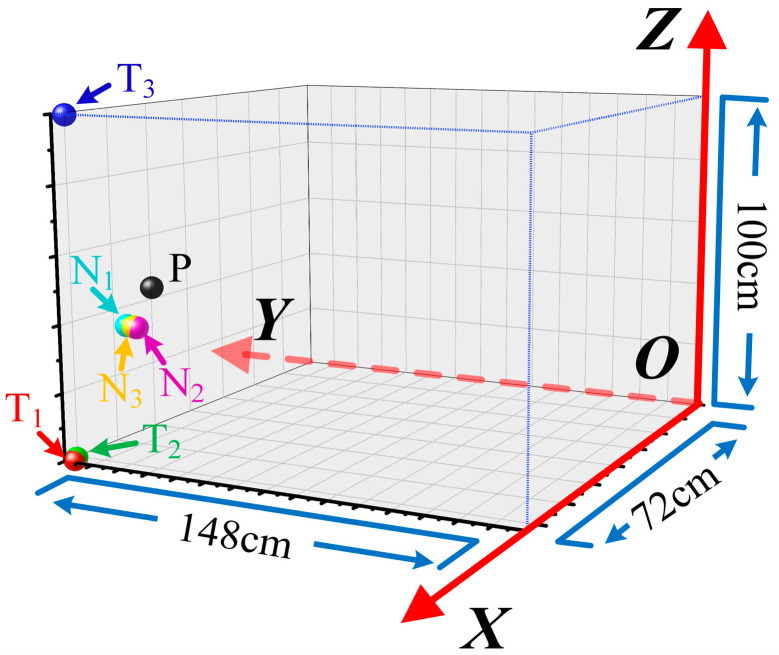
Spatial distributions of PD source localization from different methods based on six sensors.

And the spatial distributions of the localized position from these two methods are illustrated in Figure 10. Note that the localization error was defined as the spatial distance between the calculated coordinate and the real position of the PD defect. The localized results of traditional TDOA method and NLOS path correction method were T_1_ (71.91, 147.90, 0.09), T_2_ (71.50, 148.00, 0.44), T_3_ (71.20, 148.00, 99.58) and N_1_ (71.58, 127.69, 41.34), N_2_ (71.91, 123.70, 41.17), N_3_ (72.00, 124.77, 41.40), respectively. It can be seen that the calculated errors of the traditional TDOA method are relatively larger, and the errors obtained from all three experiments exceed 50 cm, and the spatial distributions of the traditional TDOA method are random and scattered, which means the computational process is unstable, leading to significant deviations in the outcomes of each calculation. When it comes to the NLOS path correction method, the spatial positions of the localization results from each experiment almost overlap. It indicates that the calculated stability of this method is improved. Further, the localized precision of the NLOS method also significantly surpasses that of the traditional TDOA method. The range of calculated errors is only within 10.88~12.01 cm.

### 4.2. Influence of the Sensors’ Number on Calculated Accuracy of the Proposed NLOS Path Correction Method

In order to certify the superiority of the number of sensors adopted in this study, here the influence of the number of sensors on calculated accuracy was discussed based on the proposed NLOS path correction method.

The measured signals were extracted from Experiment 1, and the localization results are shown in Table 2. As for the condition of four and five sensors arrangement, the range of localization error was 33.62 cm~98.55 cm and 22.39~96.76 cm. In addition, with sensors’ number increasing, the average error decreased from 60.60 cm to 10.88 cm. Here, the improving rate of calculated accuracy is defined as

(13) $$\eta =\frac{E_{n}}{E_{m}-E_{n}}\times 100\%$$

Here, *η* represents the improving rate of calculated accuracy, *E_m_* and *E_n_* represent the localization error when the number of sensors was *m* and *n*, respectively.

Based on (13), as seen, when the sensors’ number increased from four to five, the improvement rate of calculated accuracy was 14.72%. And when sensors’ number increased to six, compared to the results with four and five sensors, the localization errors were improved by 82.04% and 78.94%, respectively. It can be seen that the arrangement with 6 sensors has a more significant effect on the improvement of calculated accuracy.

**Figure 11:**
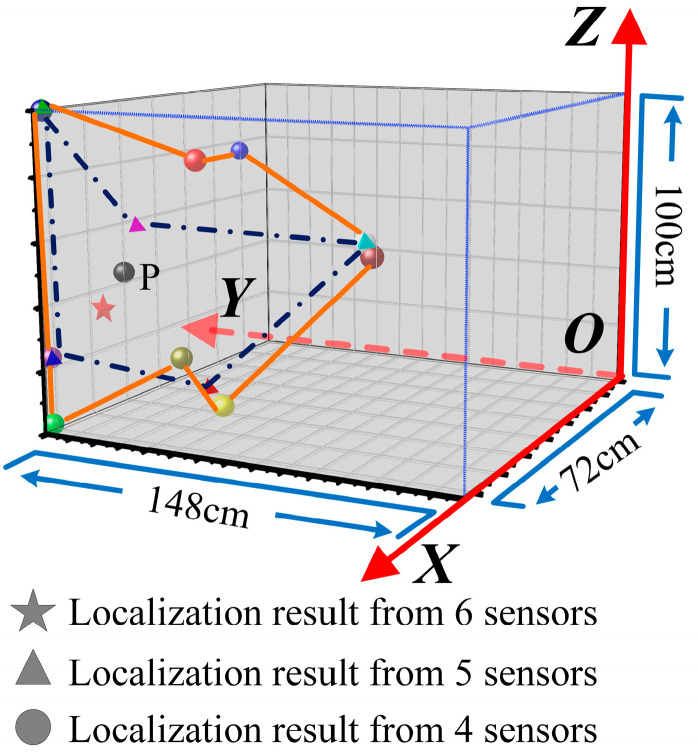
Spatial distributions of PD source localization based on NLOS path correction method with different sensor numbers.

The spatial distribution of a different number of sensors is shown in Figure 11. The localization results under all conditions of sensor numbers are centered around the actual position of the PD source, indicating that the proposed NLOS path correction method has a certain degree of calculated accuracy. By connecting the localization points with the same sensor number arrangement, a localization spatial region can be formed. It is found that the area of the spatial region from four sensors localization was larger than that of five sensors, which means that increasing the sensor number helps to enhance the stability of the localization calculation, obtaining a smaller dispersion and higher calculated accuracy in the localization results. When the sensor number increased to six, the calculated result became closer to the actual position of the PD source. In addition, some of the calculated results of four and five sensors were close to the edge of the transformer casing, which may be due to the different positions of the involved sensors during the computation process, and the influence of NLOS of UHF signals varies greatly under different sensor arrangements. However, the experiments with six sensors possess enough sensors to arrange the sensors around the discharge source, thus significantly reducing the negative impact of NLOS on calculations.

## 5. Conclusions

In this paper, a PD source localization method considering the NLOS propagation phenomenon of electromagnetic waves in a transformer was proposed, i.e., the NLOS path correction method. The conclusions are summarized as follows:

(1) The proposed PD source localization method can correct the NLOS localization errors of electromagnetic wave signals.

Moreover, the localization accuracy can be improved by increasing the number of UHF sensors.

(2) Compared to the traditional TDOA method, which employs only four UHF sensors, using more than four UHF sensors can reduce the localization error to within 30 cm, but the improvement of localization accuracy becomes insignificant when the UHF sensor number exceeds six.

(3) The experimental verification was conducted based on a testing transformer (YOD-500/35) with six UHF sensors installed inside the transformer. The results showed that, compared to the traditional TDOA method, the proposed NLOS path correction method enables us to localize the PD source position more accuracy, and the calculated process of NLOS path correction method seemed to be more stable, leading to its calculated spatial position becoming more concentrated and even tended to overlap and the range of calculated errors only within 10.88 cm~12.01 cm. In addition, compared to the results with four and five sensors, the calculated accuracy of the six-sensor arrangement was improved by 82.04% and 78.94%, respectively. The localization with six sensors is more accurate and stable, significantly reducing the negative impact of NLOS on calculations.

The validation of this study is now completed on a 35 kV test transformer. The platform is small in size, so all sensors are able to capture localized discharge signals with a high signal-to-noise ratio. It should be noted that the attenuation of UHF signals in large transformers in real substations may be higher than in this experimental environment. Therefore, the effectiveness of the method proposed in this paper is strictly dependent on the high SNR signal reception conditions. Further validation of its engineering applicability in more complex scenarios is needed in the future.

However, the transformer size in this study was small, so all sensors were able to capture localized discharge signals with high signal-to-noise ratios. It should be noted that the attenuation of UHF signals in large transformers in real substations may be higher than in this experimental environment. Therefore, the effectiveness of the method proposed in this paper is strictly dependent on the high SNR signal reception conditions. Further validation of its engineering applicability in more complex scenarios is needed in the future.

## Figures and Tables

**Table 1:** The comparison between different localization methods.

No.	Traditional TDOA Method	NLOS Path Correction Method
Experiment 1	Localization result (cm): T1 (71.91, 147.90, 0.09) Calculated error (cm): 54.17	Localization result (cm): N1 (71.58, 127.69, 41.34) Calculated error: 10.88
Experiment 2	Localization result (cm): T2 (71.50, 148.00, 0.44) Calculated error (cm): 53.83	Localization result (cm): N2 (71.91, 123.70, 41.17) Calculated error (cm): 12.01
Experiment 3	Localization result (cm): T3 (71.20, 148.00, 99.58) Calculated error (cm): 53.82	Localization result (cm): N3 (72.00, 124.77, 41.40) Calculated error (cm): 11.54

**Table 2:** Localization results of NLOS path correction method based on different sensor numbers.

Sensor Number	The Sensors Participating in Calculations	Localization Result (cm)	Calculation Error (cm)	Average Error (cm)
4	S1, S2, S3, S4	(72.00, 90.38, 87.51)	53.58	60.60
	S1, S2, S3, S5	(72.00, 148.00, 2.68)	51.85	
	S1, S2, S3, S6	(12.00, 148.00, 77.00)	62.75	
	S1, S2, S4, S5	(72.00, 33.00, 67.00)	96.76	
	S1, S2, S4, S6	(72.00, 33.00, 67.00)	96.76	
	S1, S2, S5, S6	(72.00, 82.00, 16.00)	57.63	
	S1, S3, S4, S5	(72.00, 28.87, 96.99)	38.17	
	S1, S3, S4, S6	(72.00, 148.00, 100.00)	54.30	
	S1, S3, S5, S6	(72.00, 148.00, 23.89)	33.62	
	S1, S4, S5, S6	(72.00, 30.66, 63.74)	98.55	
5	S1, S2, S3, S4, S5	(72.00, 87.63 20.34)	50.58	51.68
	S1, S2, S3, S4, S6	(72.00, 148.00, 100.00)	54.30	
	S1, S2, S3, S5, S6	(72.00, 148.00, 22.94)	34.37	
	S1, S2, S4, S5, S6	(72.00, 33.00, 67.00)	96.76	
	S1, S3, S4, S5, S6	(71.37, 114.16,66.42)	22.39	
6	S1, S2, S3, S4, S5, S6	(71.58, 127.69, 41.34)	10.88	10.88

## Data Availability

Data will be made available from the corresponding author upon reasonable request.
